# Characteristics and outcomes of Stanford type A aortic dissection patients with severe post-operation hyperbilirubinemia: a retrospective cohort study

**DOI:** 10.1186/s13019-020-01243-7

**Published:** 2020-07-28

**Authors:** Xiaolan Chen, Ming Bai, Lijuan Zhao, Yangping Li, Yan Yu, Wei Zhang, Feng Ma, Shiren Sun, Xiangmei Chen

**Affiliations:** 1 The Nephrology Department of Xijing Hospital, the Fourth Military Medical University, No. 127 Changle West Road, Xi’an, 710032 Shaanxi China; 2 State Key Laboratory of Kidney Disease, Department of Nephrology, Chinese People’s Liberation Army General Hospital and Military Medical Postgraduate College, 28th Fuxing Road, Beijing, 100853 China

**Keywords:** Stanford type a aortic dissection, Hyperbilirubinemia, Acute kidney injury, Continuous renal replacement therapy

## Abstract

**Background:**

Hyperbilirubinemia is one of the common complications after cardiac surgery and is associated with increased mortality. However, to the best of our knowledge, the reports on clinical significance of postoperative severe hyperbilirubinemia in Stanford type A aortic dissection (AAD) patients were limited.

**Methods:**

Patients who underwent surgical treatment for AAD in our center between January 2015 and December 2018 were retrospectively screened. In-hospital mortality, long-term mortality, acute kidney injury (AKI), and the requirement of continuous renal replacement therapy (CRRT) were assessed as endpoints. Univariate and multivariate regression models were employed to identify the risk factors of these endpoints.

**Results:**

After screening, 271 patients were included in our present study. Of the included patients, 222 (81.9%) experienced postoperative AKI, and 50 (18.5%) received CRRT. The in-hospital mortality was 30.3%. The 1-year, 2-year, and 3-year cumulative mortality were 32.9, 33.9, and 35.3%, respectively. Multivariate Logistic regression analysis indicated that age (*P* < 0.033), AKI stage 3 (*P* < 0.001), the amount of blood transfusion after surgery (*P* = 0.019), mean arterial pressure (MAP) in the first postoperative day (*P* = 0.012), the use of extracorporeal membrane oxygenation (ECMO) (*P* = 0.02), and the peak total bilirubin (TB) concentration (*P* = 0.023) were independent risk factors of in-hospital mortality. The optimal cut-off value of peak TB on predicting in-hospital mortality was 121.2 μmol/L. Patients with post-operation TB ≥ 121 μmol/L was associated with worse long-term survival as well.

**Conclusions:**

Severe post-operation hyperbilirubinemia is a common clinical situation in patients had AAD repair. In AAD patients with severe post-operation hyperbilirubinemia, older age, lower MAP, increased blood transfusion, stage 3 AKI, the use of ECMO, and the increased peak TB lead to increase in-hospital mortality.

## Introduction

Aortic dissection (AD) is an acute life-threatening condition with a prevalence of about 3/100,000 per year. The International Registry of AD revealed that 67% of AD patients presented with Stanford type A aortic dissection (AAD), which was characterized as the involvement of the ascending aorta. And, approximately 86% of AAD patients required swift open cardiac surgery to avoid fatal complications such as aortic rupture and cardiac tamponade [1–3]. In spite of the improvement in medical management and surgical technique, the averaged in-hospital mortality of AAD was as high as 14% [4].

Hyperbilirubinemia is a common severe complication after cardiac operation [5]. The reported incidence of post-operation hyperbilirubinemia varied widely (10 to 57%) and related to the severity of cardiac diseases and the type of cardiac surgery [5–8]. Additionally, hyperbilirubinemia was reported increased major adverse events and in-hospital and 30-day mortality for patients with cardiac surgery including aortic dissection surgery [9, 10]. Nevertheless, the effects of hyperbilirubinemia on patient prognosis with different types of surgery were heterogeneous [6, 7, 11–13]. For patients undergoing cardiac surgery with cardiopulmonary bypass (CPB), recent study suggested that severe hyperbilirubinemia (5 times the normal upper limit) instead of mild bilirubin significantly increased patient mortality and a maximum bilirubin of 25.5 mg/dl was associated with 99% mortality [14]. Mild or moderate hyperbilirubinemia might be associated with hemolysis, cardiotomy suction, gaseous micro-emboli, and blood transfusions during CPB, which were temporary and revisable. However, severe hyperbilirubinemia could induce oxidative stress and cell apoptosis, which cause respiratory failure, thrombocytopenia, and even neurological dysfunction, and consequently promote multiple organ dysfunction syndrome (MODS) and increase patient in-hospital mortality. However, the mortality of patients with severe post-operation hyperbilirubinemia remained significant divergence. Some of these patients recovered within weeks, others progressed to MODS and resulted in short-term death. The description of the characteristics, outcome, and risk factors of in-hospital and long-term mortality could help clinicians on the understanding patients with severe post-operation hyperbilirubinemia and the estimation of the patient prognosis, which was important for patient consulting and decision making. Additionally, the pathogenesis, disease severity classification, prognosis, and operation method are different between different cardiac diseases. The inclusion of all kinds of cardiac surgery in one study most likely would reduce the specificity and repeatability of the conclusions. Up to now, the reports on the characteristics and outcomes of AAD surgery patients with severe post-operation hyperbilirubinemia are limited.

Therefore, the purpose of our present study is to describe the clinical characteristics and to investigate the risk factors of mortality for AAD surgery patients who had severe post-operation hyperbilirubinemia.

## Patients and method

### Study design and patients selection

Our present study was retrospectively designed. Consecutive patients who underwent surgery for AD in our center between January 2015 and December 2018 were screened. AD was proven by enhanced computed tomography and defined as type A or type B according to the Stanford classification. Postoperative severe hyperbilirubinemia was defined as the occurrence of serum TB concentration ≥ 85.5 μmol/l (5 times the normal upper limit) in any measurement during the hospital staying after AAD surgery. Patients were excluded if they had any of the following conditions: (1) serum TB concentration < 85.5 μmol/l, (2) age < 18 years; (3) Stanford type B aortic dissection; (4) the occurrence of severe hyperbilirubinemia before surgery; (5) severe hyperbilirubinemia caused by the reoperation during hospitalization. The ethics committee of the Xijing hospital approved this retrospective study and waived the requirement of informed consent for the use of patients’ medical data.

### Surgical procedure

The use of surgical techniques has developed during the study period. However, the basic principles for repair of AAD have no change, including (1) prompt establishment of CPB, (2) resection of the primary entry site by open distal anastomosis under deep hypothermic circulatory arrest and selective anterograde cerebral perfusion, (3) preservation of the aortic valve whenever possible, and (4) aortic arch replacement in patients with an entry site located in or extending into the aortic arch. Aortic root replacement with a composite prosthesis and reimplantation of the coronary arteries by the modified Bentall technique was performed in patients with conspicuous dilatation of the aortic root.

### Data collection

Demographic data, comorbidities, and operation details were retrieved from our hospital’s electronic medical record system. All the routine laboratory data was recorded before operation (the nearest to the time of surgery) and in the post-operation period. The severity of illness before and after surgery was assessed by using the acute physiology and chronic health evaluation II (APACHE II), sequential organ failure assessment (SOFA) score, and model for end-stage liver disease (MELD) score. Urine output was recorded every day after the surgery.

### Outcomes and definition

Post-operation outcomes including the amount of blood transfusion, mechanical ventilation time, the use of extracorporeal membrane oxygenation (ECMO), intra-aortic balloon pump (IABP), AKI, the usage of CRRT, bilirubin adsorption (BA) or plasma exchange (PE), and vasoactive agent, the duration of hospitalization, ICU stay time, and in-hospital mortality. For those patients who were alive on discharge, telephone survey was performed to obtain the patients long-term outcome.

Kidney Disease Improving Global Outcomes (KDIGO) criteria [15] based on SCr or urine output was employed to diagnose and grade AKI. The latest SCr concentration before surgery was defined as preoperative SCr concentration. The decision to start CRRT was made at the discretion of the attending nephrologist. Main indications for starting CRRT were progressive AKI, fluid overload, hyperkalemia, and severe metabolic acidosis.

### Statistical analysis

Continuous variables are presented as mean ± standard deviation. Categorical variables are presented as frequencies with percentages. To evaluate the differences between groups, the independent sample *t-*test was used for continuous variables, whereas the chi-square test or Fisher’s exact test was used for categorical variables. Factors significantly associated with these endpoints in univariate analysis were included in the multivariate logistic regression analysis or Cox proportional hazard analysis to identify the independent risk factors. Accumulated survival proportion was estimated with the Kaplan-Meier method, and the between-group differences of survival proportion were assessed using the log-rank test. Area under the receiver operating characteristic curve (AUC-ROC) was calculated to assess peak TB concentration on the ability to detect in-hospital mortality. Youden index was used for assessment of optimal cut-off values. For all analyses, all statistical tests were 2-sided, and a *P*-value < 0.05 was considered as statistically significant. Data were analyzed using SPSS version 22.0 software (SPSS, Inc., Chicago, IL, USA).

## Result

### Patient characteristics

**Fig. 1:**
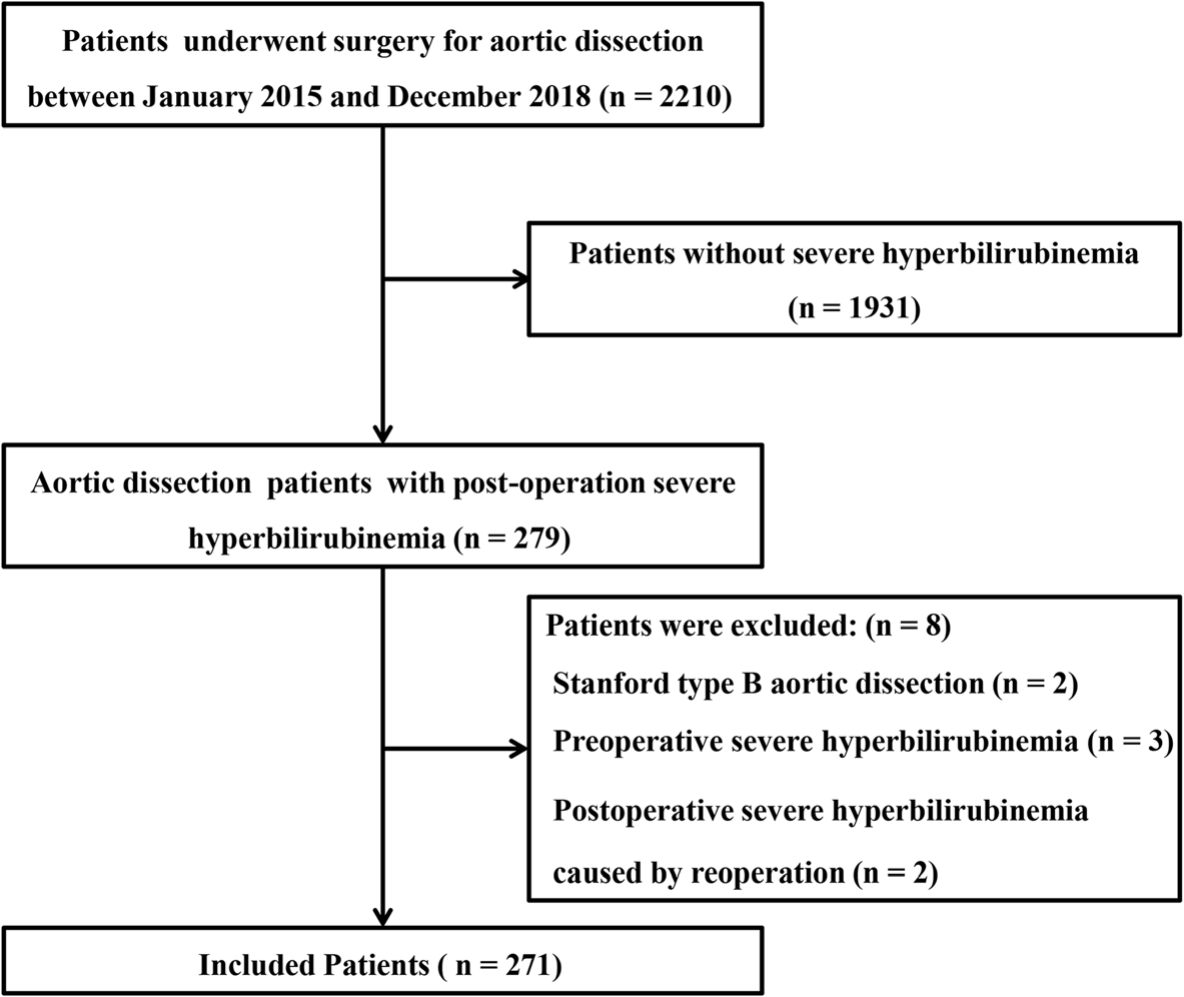
Patient inclusion flow chart

Of the 2210 screened patients, 279 patients (12.6%) developed severe post-operation hyperbilirubinemia. Of these patients, 2, 3, and 3 were excluded because of Stanford type B aortic dissection, preoperative TB ≥ 85.5 μmol/l, and severe post-operation hyperbilirubinemia caused by reoperation during this hospitalization, respectively. Ultimately, 271 AAD surgery patients with severe post-operation hyperbilirubinemia were included in our present study (Fig. 1).

**Fig. 2:**
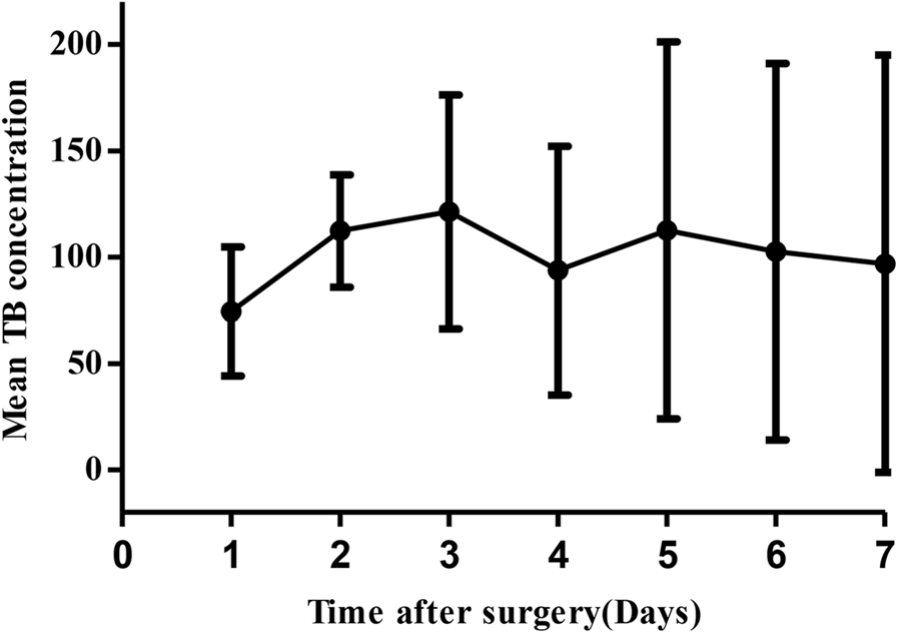
The averaged serum total bilirubin concentration during the postoperative seven days

The baseline characteristics of the included patients were summarized in Table 1. There were 223 male and 48 female, and the mean age of the patients was 49.1 ± 11.0 years. AAD surgery involved the ascending aortic in 83.8% patients, aortic valve in 70.7% patients, aortic arch in 86% patients, and coronary artery in 17% patients. Preoperative TB concentration was 25.0 ± 15.0 μmol/l. The mean onset time of severe hyperbilirubinemia was 2.8 ± 1.3 days after AAD surgery. The mean peak serum TB concentration was 150.9 ± 93.0 μmol/l and the averaged time to peak TB concentration was 3.8 ± 3.0 days after AAD surgery. The change of TB concentration during the 7 days after surgery was showed in Fig. 2. The median follow-up time was 18.63 (0.5–55.9) months. And, 3 patients were lost to follow-up. The time of lost to follow-up was 16-, 95-, and 365-day after the surgery, respectively.

**Table 1:** Baseline characteristics of the included patients

Variables	Value
Preoperative	
Age, mean ± SD (years)	49.1 ± 11.0
Male, n (%)	223 (82.3%)
Co-morbidity	
Hypertension, n (%)	158 (58.3%)
Diabetes, n (%)	3 (1.1%)
Cerebrovascular disease, n (%)	16 (5.9%)
Previous cardiac surgery, n (%)	8 (2.9%)
APACHEII score, mean ± SD	9.2 ± 3.2
MELD score, mean ± SD	9.2 ± 4.1
SOFA score, mean ± SD	2.1 ± 1.5
MAP, mean ± SD (mm/Hg)	90.5 ± 12.9 (70–105)
TB, mean ± SD (μmol/l)	↑25.0 ± 15.0 (3.4–20.5)
CB, mean ± SD (μmol/l)	↑7.8 ± 8.1 (0.0–6.8)
WBC, mean ± SD (10^9^/l)	↑11.7 ± 5.0 (3.5–9.5)
Hb, mean ± SD (g/l)	136.1 ± 19.9 (130–175)
PLT, mean ± SD (10^9^ /l)	↑157.2 ± 68.5 (125–350)
SCr, mean ± SD (μmol/l)	↑111.1 ± 38.3 (57–111)
PT, mean ± SD (s)	12.0 ± 2.2 (9.8–12.1)
Intraoperative	
Type of operation	
Ascending aorta, n (%)	227 (83.8%)
Aortic valve	191 (70.7%)
Aortic arch, n (%)	233 (86%)
Coronary artery, n (%)	46 (17%)
Operation duration, mean ± SD (h)	6.8 ± 1.6
CPB time, mean ± SD (min)	226.5 ± 60.9
ACC time, mean ± SD (min)	102.2 ± 28.0
Arrest time, mean ± SD (min)	25.1 ± 14.4
The amount of blood transfusion (U)	18.8 ± 10.6
Postoperative	
APACHE II score	16.9 ± 2.5
SOFA score	12.1 ± 2.6
Meld score	18.6 ± 4.4
AST, mean ± SD (U /L)	↑347.6 ± 1387.2 (9–50)
ALT, mean ± SD (U/L)	↑164.4 ± 526.6 (9–50)
TB, mean ± SD (μmol /l)	↑189.1 ± 74.6 (3.4–20.5)
CB, mean ± SD (μmol /l)	↑110.7 ± 37.9 (0.0–6.8)
WBC, mean ± SD (10^9^/l)	↑12.9 ± 4.8 (3.5–9.5)
Hb, mean ± SD (g/l)	112.9 ± 16.6 (130–175)
PLT, mean ± SD (10^9^ /l)	↓99.8 ± 49.5 (125–350)
SCr, mean ± SD (μmol/l)	↑162.8 ± 71.8 (57–111)
PT, mean ± SD (s)	13.6 ± 2.9 (9.8–12.1)
Peak TB level, mean ± SD (μmol/l)	150.9 ± 93.0
Peak TB level > 171 μmol/l, n (%)	55 (20.4%)
Peak TB level > 340 μmol/l, n (%)	12 (4.4%)
Time to peak TB level, mean ± SD (d)	3.8 ± 3.0
Time to peak TB level > 5 d, n (%)	47 (17.4%)

*ACC* Aortic cross clamp, *APACHEII* Acute physiology and chronic health evaluation II, *CB* Conjugated bilirubin, *CPB* Cardiopulmonary bypass, *Hb* Hemoglobin, *MAP* Mean arterial pressure, *MELD* Model for end-stage liver disease, *min* minute, *PLT* Platelet, *PT* Prothrombin time, *SCr* Serum creatinine, *SD* Standard deviation, *SOFA* Sequential organ failure assessment, *TB* Total bilirubin, *WBC* White blood cell

Parentheses provide normal ranges for the depicted values; ↑ indicate variables greater than normal range and ↓ indicate variables less than normal range

### Postoperative AKI

Of the 271 included patients, 222 patients (82.1%) had AKI after AAD surgery, of which 102 (40.2%), 34 (12.5%), and 84 (29.2%) were stage 1, stage 2, and stage 3 AKI (Table 2), respectively. The results of univariate and multivariate logistic regression are presented in Table 3. In the univariate analysis, age, hypertension, preoperative SCr concentration, APACHE II score, and MELD score, operation time, and CPB time were associated with the occurrence of postoperative AKI. Multivariate analysis indicated that increased age (OR 1.056, 95%CI 1.026–1.088, *P* < 0.001), higher preoperative SCr concentration (OR 1.026, 95%CI 1.010–1.043, *P* = 0.002), and prolonged CPB time (OR 1.007, 95%CI 1.000–1.013, *P* = 0.042) were identified as independent risk factors of AKI in AAD surgery patients with severe post-operation hyperbilirubinemia.

**Table 2:** The major outcomes of the included patients

Variables	Value
In-hospital mortality, n (%)	82 (30.3%)
Cause of death	
Multiple organ failure, n (%)	40 (48.8%)
Heart failure, n (%)	35 (42.7%)
Hemorrhagic shock, n (%)	2 (2.4%)
Sepsis, n (%)	5 (6.1%)
Reoperation, n (%)	17 (6.3%)
AKI, n (%)	222 (81.9%)
Stage of AKI	
Stage 1, n (%)	102 (40.2%)
Stage 2, n (%)	34 (12.5%)
Stage 3, n (%)	84 (29.2%)
Use of CRRT, n (%)	50 (18.5%)
Use of PE/BA, n (%)	11 (14.1%)
Use of IABP, n (%)	1 (0.4%)
Use of ECMO, n (%)	7 (2.6%)
Use of vasoactive agent, n (%)	173 (63.8%)
Mechanical ventilation time, mean ± SD (days)	3.3 ± 5.7
The amount of blood transfusion, mean ± SD (U)	34.8 ± 43.3
In hospital time, mean ± SD (days)	19.8 ± 10.7
ICU stay time, mean ± SD (days)	6.6 ± 6.3
Onset of severe hyperbilirubinemia, mean ± SD (days)	2.8 ± 1.3

*AKI* Acute kidney injury, *BA* Bilirubin adsorption, *CRRT* Continuous renal replacement therapy, *ECMO* Extracorporeal membrane oxygenation, *IABP* Intra-aortic balloon pump, *ICU* Intensive care unit, *PE* Plasma exchange

**Table 3:** Logistic regression analysis for postoperative AKI and CRRT

Characteristic	Univariate logistic regression		Multivariate logistic regression	
	OR (95%CI)	***P*** value	OR (95%CI)	***P*** value
**Postoperative AKI**				
Age	1.061 (1.032–1.092)	< 0.001	1.056 (1.026–1.088)	< 0.001
Hypertension (yes/no)	2.766 (1.469–5.208)	0.002		
Preoperative				
SCr	1.030 (1.014–1.047)	< 0.001	1.026 (1.010–1.043)	0.002
APACHE II score	1.399 (1.213–1.615)	< 0.001		
MELD score	1.130 (1.035–1.233)	0.007		
Operation duration	1.346 (1.089–1.633)	0.006		
CPB time	1.008 (1.001–1.014)	0.015	1.007 (1.000–1.013)	0.042
**Postoperative CRRT**				
Male (yes/no)	4.006 (1.192–13.462)	0.025		
Preoperative				
SCr	1.011 (1.003–1.018)	0.004	1.009 (1.002–1016)	0.017
APACHE II score	1.333 (1.033–1.244)	0.008		
MELD score	1.083 (1.007–1.164)	0.032		
ACC time	1.011 (1.000–1.022)	0.049		

*ACC* Aortic cross clamp, *AKI* Acute kidney injury, *APACHEII* Acute physiology and chronic health evaluation II, *CPB* Cardiopulmonary bypass, *CRRT* Continuous renal replacement therapy, *MELD* Model for end-stage liver disease, *SCr* Serum creatinine

### Postoperative CRRT

Of the included patients, 50 (18.5%) patients received CRRT after AAD surgery. Univariate analysis indicated that male, preoperative SCr concentration, APACHE II score, MELD score, and aortic cross-clamp (ACC) time were associated with the need for CRRT. In the multivariate logistic regression analysis, only preoperative SCr concentration (OR 1.011, 95%CI 1.002–1016, *P* = 0.003) was identified as an independent predictor of the acceptance of CRRT (Table 3).

### In-hospital mortality

There were 82 (30.3%) in-hospital deaths. The leading causes of death were multiple organ failure (48.8%) and heart failure (42.7%). Other causes included sepsis (6.1%) and hemorrhagic shock (2.4%) (Table 2). Univariate analysis revealed that 20 pre-operation, intra-operation, and post-operation factors were associated with in-hospital mortality. After the adjustment of the important clinical parameters, independent risk factors of in-hospital mortality identified by multivariate logistic analysis included age (OR 1.005, 95%CI 1.004–1.099, *P* = 0.033), the amount of blood transfusion (OR 1.018, 95%CI 1.003–1.033, *P* = 0.019), stage 3 AKI (OR 46.134, 95%CI 5.436–391.525, *P* < 0.001), the use of ECMO (OR 20.795, 95%CI 1.620–266.917, *P* = 0.02), and the peak TB concentration (OR 1.017, 95%CI 1.002–1.032, *P* = 0.023). Postoperative MAP tended to be a protective factor (OR 0.955, 95%CI 0.922–0.990, *P* = 0.012, Table 4, model 1).

**Table 4:** Logistic regression analysis for in-hospital mortality

Characteristic	Univariate logistic regression		Multivariate logistic regression (model 1)		Multivariate logistic regression (model 2)	
	OR (95%CI)	***P*** value	OR (95%CI)	***P*** value	OR (95%CI)	***P*** value
Age (year)	1.035 (1.009–1.062)	0.008	1.005 (1.004–1.099)	0.033	1.046 (1.002–1.093)	0.040
Male (yes/no)	2.483 (1.106–5.575)	0.027				
Preoperative						
SCr	1.009 (1.002–1.016)	0.008				
APACHE II score	1.178 (1.082–1.282)	< 0.001				
Intraoperative						
CPB time	1.007 (1.003–1.011)	0.002				
ACC time	1.012 (1.002–1.021)	0.014				
Deep hypothermic circulatory arrest time	0.997 (0.979–1.016)	0.787				
Postoperative						
MAP	0.948 (0.926–0.971)	< 0.001	0.955 (0.922–0.990)	0.012	0.963 (0.930–0.977)	0.031
Re-operation (yes/no)	3.611 (1.324–9.852)	0.012				
The total amount of blood transfusion	1.046 (1.032–1.060)	< 0.001	1.018 (1.003–1.033)	0.019	1.020 (1.005–1.034)	0.007
Mechanical ventilation time	1.533 (1.341–1.798)	< 0.001				
AKI (yes/no)	28.350 (3.842–209.203)	0.001				
Stage of AKI		< 0.001		< 0.001		< 0.001
Stage 1	5.859 (0.735–46.720)	0.095	2.424 (0.267–21.990)	0.431	2.218 (0.253–19.426)	0.472
Stage 2	10.138 (1.162–88.457)	0.036	4.560 (0.478–43.545)	0.187	3.698 (0.385–35.548)	0.257
Stage 3	165.053 (21.354–1275.729)	< 0.001	46.134 (5.436–391.525)	< 0.001	46.318 (5.666–378.663)	< 0.001
Use of CRRT (yes/no)	23.756 (10.359–54.481)	0.001				
Use of PE/BA (yes/no)	11.527 (2.432–54.631)	0.002				
Use of ECMO (yes/no)	14.842 (1.757–125.357)	0.013	20.795 (1.620–266.917)	0.02	10.130 (0.900–113.958)	0.061
Peak TB concentration	1.009 (1.005–1.012)	< 0.001	1.017 (1.002–1.032)	0.023		
Peak TB ≥121 μmol/l, (yes/no)	3.899 (2.220–6.847)	< 0.001			2.681 (1.119–6.425)	0.027
Time to peak TB concentration	1.441 (1.277–1.626)	< 0.001				
ICU stay time	1.268 (1.172–1.371)	< 0.001				

Model 1 was established by analysis of peak TB concentration; Model 2 was established by subgroup analysis of peak bilirubin concentration greater or less than 121.2 μmol/l

*ACC* Aortic cross clamp, *AKI* Acute kidney injury, *APACHEII* Acute physiology and chronic health evaluation II, *BA* Bilirubin adsorption, *CPB* Cardiopulmonary bypass, *CRRT* Continuous renal replacement therapy, *ECMO* Extracorporeal membrane oxygenation, *ICU* Intensive care unit, *MAP* Mean arterial pressure, *PE* Plasma exchange, *SCr* Serum creatinine, *TB* Total bilirubin

ROC analysis (Supplementary figure 1) identified that peak TB concentration was associated with increased mortality, and the optimal cut-off value identified by the Youden index was 121.2 μmol/l (sensitivity: 72%, specificity: 60%). And, the multivariate analysis revealed that patients with peak TB concentration ≥ 121.2 μmol/l had a significantly higher risk of in-hospital mortality (OR = 2.681, 95%CI 1.119–6.425, *P* = 0.027, Table 4, model 2), compared with the patients with peak TB concentration < 121.2 μmol/l.

### Long-term mortality

**Fig. 3:**
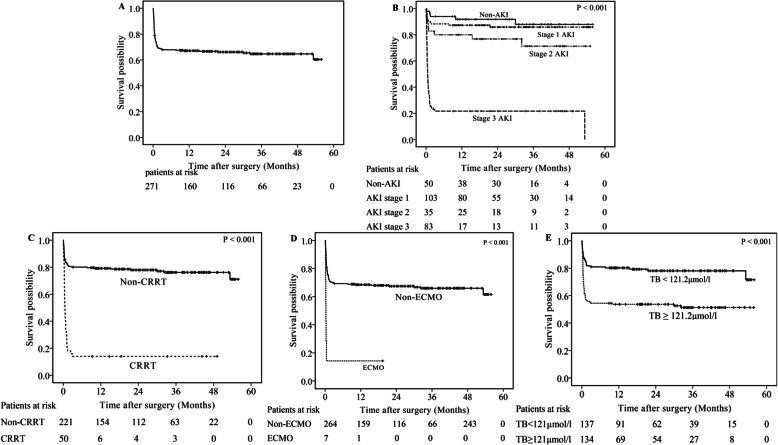
Long-term survival results of (**a**) all patients, **b** patients without AKI and those with stage 1, 2, or 3 AKI, **c** patients without the use of CRRT and those with CRRT, **d** patients without the use of ECMO and those with ECMO, **e** patients with post-operation peak TB ≥ 121.2 μmol/l and those with post-operation peak TB < 121.2 μmol/l

Of the 12 patients who died after discharge, 4 had non-AKI, 3 had stage 1 AKI, 3 had stage 2 AKI, and 2 had stage 3 AKI during their hospital stay, respectively. The accumulated 1-year, 2-year, and 3-year mortality proportions were 32.9, 33.9, and 35.3%, respectively (Fig. 3a). The risk factors of long-term mortality were presented in Table 5. Univariate analysis revealed 20 factors, including the post-operation AKI, the acceptance of CRRT and ECMO, and the peak TB concentration ≥ 121.2 μmol/l (Fig. 3b-e), were significantly related to patient long-term mortality. Multivariate COX regression analysis revealed that stage 3 AKI (HR 12.604, 95%CI 5.002–31.762, *P* < 0.001) significantly increased long-term mortality, compared with patients without AKI. The use of ECMO (HR 12.167, 95%CI 4.588–32.264, *P* < 0.001) was identified as an independent predictor of long-term mortality as well. In contrast, postoperative MAP (HR 0.979, 95%CI 0.962–0.995, *P* = 0.012) was identified as an independent protective factor of long-term mortality.

**Table 5:** COX regression analysis for long-term mortality

Characteristic	Univariate COX regression		Multivariate COX regression	
	OR (95%CI)	***P*** value	OR (95%CI)	***P*** value
Age	1.023 (1.003–1.043)	0.026		
Preoperative				
SCr	1.006 (1.002–1.010)	< 0.001		
APACHE II score	1.122 (1.060–1.188)	< 0.001		
MELD score	1.052 (1.003–1.105)	0.039		
Intraoperative				
Operation duration	1.248 (1.098–1.414)	0.001		
CPB time	1.005 (1.002–1.008)	0.001		
Postoperative				
MAP	0.971 (0.957–0.985)	< 0.001	0.979 (0.962–0.995)	0.012
The total amount of blood transfusion	1.009 (1.007–1.011)	< 0.001		
Mechanical ventilation time	1.035 (1.020–1.051)	< 0.001		
AKI (yes/no)	6.25 (2.295–17.024)	< 0.001		
Stage of AKI		< 0.001		< 0.001
Stage 1	1.404 (0.506–3.901)	0.515	1.037 (0.365–2.946)	0.946
Stage 2	2.772 (0.929–8.271)	0.068	2.236 (0.774–6.990)	0.133
Stage 3	15.071 (6.043–37.587)	< 0.001	12.604 (5.002–31.762)	< 0.001
Use of CRRT (yes/no)	6.368 (4.174–9.709)	< 0.001		
Use of PE/BA (yes/no)	3.184 (1.596–6.351)	0.001		
Use of ECMO (yes/no)	6.108 (2.638–14.139)	< 0.001	12.167 (4.588–32.264)	< 0.001
Onset of hyperbilirubinemia	1.217 (1.044–1.418)	0.012		
Peak TB concentration	1.004 (1.003–1.006)	< 0.001		
Peak TB > 121 μmol/l (yes/no)	2.595 (1.679–4.001)	< 0.001		
Peak TB > 171 μmol/l (yes/no)	2.292 (1.488–3.530)	< 0.001		
Time to peak TB concentration	1.156 (1.107–1.208)	< 0.001		
ICU stay time	1.037 (1.022–1.053)	< 0.001		

*ACC* Aortic cross clamp, *AKI* Acute kidney injury, *APACHEII* Acute physiology and chronic health evaluation II, *BA* Bilirubin adsorption, *CPB* Cardiopulmonary bypass, *CRRT* Continuous renal replacement therapy, *ECMO* Extracorporeal membrane oxygenation, *ICU* Intensive care unit, *MAP* Mean arterial pressure, *MELD* Model for end-stage liver disease, *PE* Plasma exchange, *SCr* Serum creatinine, *TB* Total bilirubin

## Discussion

It has been reported that severe hyperbilirubinemia was associated with increased mortality in patients underwent cardiac surgery [12, 14, 16]. Nevertheless, the mortality of patients with severe hyperbilirubinemia remained significant divergence. Up to now, little focus was aimed on the real risk of patients with severe hyperbilirubinemia after AAD surgery. Our presented study had several findings. Firstly, the occurrence of AKI, the requirement for CRRT, and the in-hospital mortality were higher than previous studies of patients undergoing AAD surgery without severe hyperbilirubinemia. Secondly, age, preoperative SCr concentration, and CPB time were independent risk factors for postoperative AKI, and preoperative SCr concentration was an independent risk factor for post-operation CRRT as well. Finally, the peak TB concentration, post-operation stage 3 AKI, the total amount of blood transfusion after AAD surgery, the use of ECMO, and low MAP after surgery were significantly associated with mortality.

### AAD patients with severe hyperbilirubinemia were associated with worse prognosis

The analysis of our present cohort of AAD patients with severe post-operation hyperbilirubinemia showed an overall incidence of post-operation AKI of 81.9%, a requirement for CRRT of 18.5%, and the in-hospital mortality increased from 14% in all patients to 30.3% in those with post-operative hyperbilirubinemia. In previous studies of AAD patients underwent surgical treatment without severe post-operation hyperbilirubinemia, the reported AKI incidences were ranged from 40 to 78% [17–25], the reported incidences of requirement for CRRT were ranged from 3 to 8% [21, 25], and the reported in-hospital mortalities were ranged from 15 to 26% [18, 21, 25–27]. The discrepancy most likely attributed to the fact that all of the included AAD patients in our study developed severe hyperbilirubinemia. The development of severe hyperbilirubinemia after AAD surgery related to the severity of the AAD and the severity of injury during AAD operation. Additionally, perioperative hyperbilirubinemia has been proved to be associated with postoperative AKI in patients undergoing cardiac surgeries as well [10]. In animal model, hyperbilirubinemia was proved to have pro-apoptotic effects and aggravate renal ischemia-reperfusion injury [28]. Additionally, high concentration of bilirubin could lead to inflammatory response and cell apoptosis of the brain [29], which might be another potential mechanism of the high mortality of our present cohort. Despite improvement of AAD repair technology, the employment of deep hypothermic circulatory arrest and antegrade cerebral perfusion, the improvement of CBP strategies, and the availability of advanced organ support system, all of the included AAD patients had severe post-operation hyperbilirubinemia, and 81.9% of the included patients developed AKI during their hospital staying in the present study, which increased in-hospital mortality due to the patient severity.

### Risk factors of postoperative AKI and CRRT

In our present cohort, older age, high preoperative SCr concentration, and pronged CPB time were identified as independent risk factors for AKI. Meanwhile, preoperative SCr concentration was also associated with the requirement of CRRT after AAD surgery. A recent meta-analysis of patients underwent AAD surgery [17] showed that older age was identified as an independent risk factor of AKI as well. Older age-mediated adverse renal structural and functional changes might contribute to the high development of postoperative AKI after AAD surgery in older patients. The aforementioned meta-analysis showed that preoperative SCr concentration did not correlate with postoperative AKI with significant heterogeneity (I^2^ = 72.8%). In our opinion, pre-operation elevated SCr concentration might indicate structural kidney damage or hemodynamic derangements in AAD patients, which further aggravated the development of post-operation AKI and the requirement of CRRT. Longer CPB time can lead to more hemolysis, and a longer time on the circuit can lead to changes in perfusion to the viscera and to more inflammatory action. A CPB itself might also induce hypoperfusion of abdominal organs, hypoxia or an inflammatory reaction, which causes liver and kidney damage. Therefore, surgeons most likely could reduce the AKI risk of AAD patients by the improvement of their operation strategies and the reduction of CPB time.

### Risk factors of in-hospital and long-term mortality

In previous studies, older age had been identified as an independent risk factor of mortality for patients with hyperbilirubinemia after cardiac surgery [30]. In our present study, older age was established a risk factor for in-hospital mortality for patients with hyperbilirubinemia after AAD surgery as well. Aging indicated diminished functional capacity of the liver and added to the cumulative burden in the case of developed severe hyperbilirubinemia. Additionally, different from the a recent study outlining the increased risk of mortality for patients with hyperbilirubinemia after cardiac surgery associated with time to peak bilirubin [14], our study indicated that peak TB concentration was an independent driver of in-hospital mortality for patients with severe hyperbilirubinemia after AAD surgery. And, we found out that the optimal cut-off value of peak TB on predicting in-hospital mortality was 121.2 μmol/l. This difference might be attributed to the existence of severe hyperbilirubinemia after AAD surgery and the limitation of small sample size. The high concentration of bilirubin leads to inflammatory response or cell apoptosis in the brain [29]. Cui et al. [31] showed that bilirubin induced lung edema and injury by inducing the apoptosis of alveolar epithelial cells. From the above, severe hyperbilirubinemia induces oxidative stress and apoptosis in many organs, which is associated with poor outcomes in patients with AAD surgery.

In a study for patients with jaundice after open heart surgery, Chu et al. [32] reported patients with more severe hyperbilirubinemia or delayed serum peak TB concentration might be significantly associated with more transfused blood and hypotension. Increased amount of blood transfusions contributed to hemolysis, and hypotension would reduce hepatic perfusion [33], which both caused an increased bilirubin load. This might be one of the potential mechanisms that the number of transfused blood units and lower MAP increased mortality in our present study. Previous studies suggested that mechanisms underlying the development of post-operation late peak TB concentration differed from those of the early peak bilirubin concentration. The immediate development of post-operation peak TB concentration and rapid decline thereafter reflected the transient damaging effects by CPB surgery, whereas late development of post-operation peak TB concentration was a consequence of hepatic dysfunction caused by persistent cardiac failure or sepsis [5, 7]. In the present study, multiple organ failure, cardiac failure, and sepsis were observed among hospitalized deaths as well. Therefore, attention should be paid to the monitoring of heart failure and optimizing hemodynamics after AAD surgery to prevent further deterioration. Furthermore, identifying and implementing effective risk reduction strategies is needed. Molecular adsorbent recirculation system, prometheus therapy, fractionated plasma separation and adsorption have shown promise in reducing bilirubin concentration [30, 34], but the ideal timing of blood purification initiation after cardiac surgery remains uncertain and further study in this area is needed.

AKI had been incorporated into a risk tool to predict early mortality for patients underwent AAD surgery [17]. Our results added to these findings and demonstrated stage 3 AKI markedly increased both in-hospital and long-term mortality. We found patients who had heart failure after cardiac surgery developed hyperbilirubinemia and AKI. Heart failure could cause reduced systemic blood flow, which leaded to inappropriate oxygen delivery and energy deficit. These caused kidney and liver damage, Additionally, severe hyperbilirubinemia aggravated renal ischemia-reperfusion injury and increased the incidence of severe AKI [28]. Additionally, we found most patients died of multiple organ failure or heart failure, which might be one of potential mechanism that AKI and severe hyperbilirubinemia were associated with high mortality. Additionally, our study also showed in-hospital and long-term mortality was independently predicted by the use of ECMO. Hemolysis is a common complication in ECMO support [35]. Pump head or oxygenator thrombosis and excessive pump speed (typically greater than 3000 rpm) which may destroy blood red cells [36], which in turn is important factor in the development of hyperbilirubinemia. Therefore, more careful postoperative management is needed to improve prognosis.

### Study limitation

Our present study was a retrospective clinical research from a single institution and had some limitations. First, SCr concentration on admission was regard as preoperative renal function. However, some patients might already have AKI on admission. As a result, the number of patients with AKI may be underestimated. Second, the number of major adverse events including the use of ECMO, plasma exchange and bilirubin adsorption was small, which will be likely to reduce the statistical power for risk factor analysis. Finally, the renal prognosis was not regular followed up after the hospital discharge, which is important for the evaluation of the renal outcome. Therefore, further prospective multicenter studies with larger samples are needed to obtain stronger evidences.

## Conclusion

Severe post-operation hyperbilirubinemia is a common clinical presentation in AAD surgery patients. Severe post-operation hyperbilirubinemia AAD patients with older age, lower MAP, increased blood transfusion, stage 3 AKI, the use of ECMO, and the increased peak TB had higher risk of in-hospital mortality. These patients most likely need more intensive monitoring.

## Supplementary information

**Additional file 1: Figure S1.** Receiver operator curve (ROC) of peak TB concentration predicting in-hospital mortality (area under the curve: 0.68, 95% CI: 0.614–0.758).

## Data Availability

The datasets used or analyzed during the current study are available from the corresponding author on reasonable request.

## Abbreviations

AAD: Stanford type A aortic dissection
AKI: Acute kidney injury
ACC: Aortic cross clamp
APACHEII: Acute physiology and chronic health evaluation II
BA: Bilirubin adsorption
CPB: Cardiopulmonary bypass
CRRT: Continuous renal replacement therapy
ECMO: Extracorporeal membrane oxygenation
IABP: Intra-aortic balloon pump
ICU: Intensive care unit;
MAP: Mean arterial pressure
MELD: Model for end-stage liver disease
PE: Plasma exchange
SCr: Serum creatinine
SOFA: Sequential organ failure assessment
TB: Total bilirubin
