# Supplementary figures and images for: Characteristics and outcomes of Stanford type A aortic dissection patients with severe post-operation hyperbilirubinemia: a retrospective cohort study
Source: J Cardiothorac Surg. 2020 Jul 28;15:195. doi: 10.1186/s13019-020-01243-7 (PMC7388495; doi:10.1186/s13019-020-01243-7)

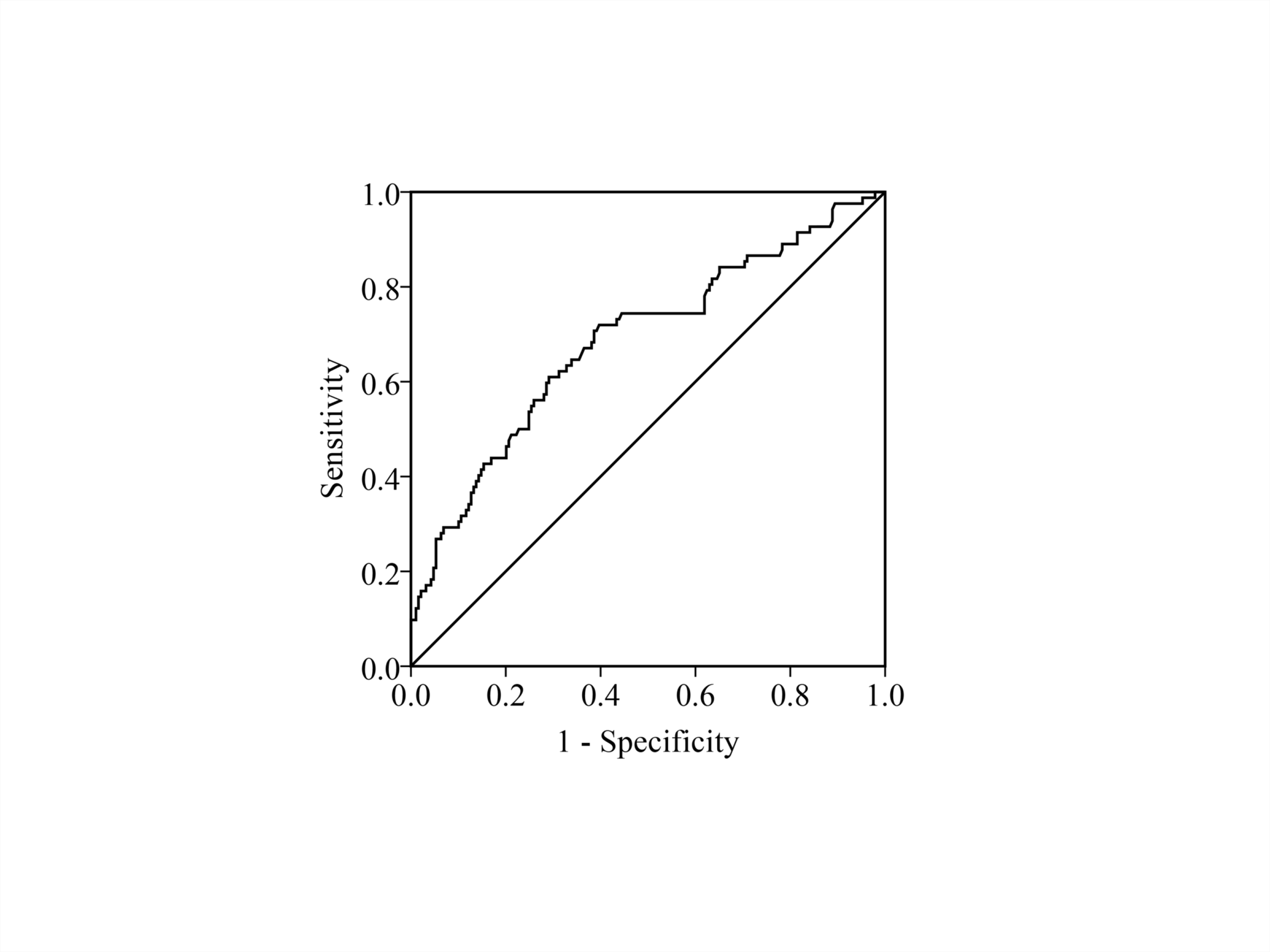

Supplement: Supplementary file 1 — Additional file 1: Figure S1. Receiver operator curve (ROC) of peak TB concentration predicting in-hospital mortality (area under the curve: 0.68, 95% CI: 0.614–0.758). [file 13019_2020_1243_MOESM1_ESM.tif]
